# Subjective ratings and emotional recognition of children’s facial expressions from the CAFE set

**DOI:** 10.1371/journal.pone.0209644

**Published:** 2018-12-27

**Authors:** Marília Prada, Margarida V. Garrido, Cláudia Camilo, David L. Rodrigues

**Affiliations:** Department of Social and Organizational Psychology, Instituto Universitário de Lisboa (ISCTE-IUL), CIS - IUL, Lisboa, Portugal; Temple University, UNITED STATES

## Abstract

Access to validated stimuli depicting children’s facial expressions is useful for different research domains (e.g., developmental, cognitive or social psychology). Yet, such databases are scarce in comparison to others portraying adult models, and validation procedures are typically restricted to emotional recognition accuracy. This work presents subjective ratings for a sub-set of 283 photographs selected from the Child Affective Facial Expression set (CAFE [1]). Extending beyond the original emotion recognition accuracy norms [2], our main goal was to validate this database across eight subjective dimensions related to the model (e.g., attractiveness, familiarity) or the specific facial expression (e.g., intensity, genuineness), using a sample from a different nationality (*N* = 450 Portuguese participants). We also assessed emotion recognition (forced-choice task with seven options: anger, disgust, fear, happiness, sadness, surprise and neutral). Overall results show that most photographs were rated as highly clear, genuine and intense facial expressions. The models were rated as both moderately familiar and likely to belong to the in-group, obtaining high attractiveness and arousal ratings. Results also showed that, similarly to the original study, the facial expressions were accurately recognized. Normative and raw data are available as supplementary material at https://osf.io/mjqfx/.

## Introduction

Children communicate positive and negative emotions through multiple channels, namely: vocalizations, gestures, body postures, body movements and facial expressions (for a review, see [3]). Traditionally, research has focused on the latter. Not only do facial expressions signal the children’s emotional state, but they can also evoke behavioral motives (e.g., motivation to nurture) in the observers (for a review, see [4]). Importantly, parent-child interaction and parental mental health may be predicted by how accurately the children’s emotional expression is perceived (for a review, see [5]).

The availability of validated children’s facial expressions databases is important for several research domains. However, in contrast to databases depicting adult models, such databases are still scarce and usually are only validated for the accuracy of emotional recognition. The goal of the current work was to extend the available norms for the Child Affective Facial Expression (CAFE; [2]), a database that exclusively includes photographs depicting facial expressions of children. Besides emotion recognition, for each stimulus, we also assessed a set of eight subjective evaluative dimensions concerning the model (familiarity, attractiveness, arousal, and in-group belonging) and the expression (valence, clarity, intensity, and genuineness) being portrayed. These additional subjective ratings provide important information that further extends the usefulness of the stimuli set. Specifically, it enables the selection of stimuli through a combination of criteria (e.g., happy faces controlled for attractiveness; fear faces varying in intensity).

Static human face stimuli are the most frequently used type of material in emotion recognition and detection studies, and have been relying on both behavioral (e.g., forced-choice labeling of emotions; matching task) and non-behavioral methodologies (e.g., functional and structural MRI, EEG; for a review, see [6]).

In studies with children populations these materials are often used to investigate how (and at what age) children are able to understand and identify emotional faces (e.g., [7], for reviews, see [8,9]), or to characterize their affective reactions to emotional facial expressions (e.g., [10]). Importantly, children who are better at recognizing emotions in others also tend to be successful in several socioemotional areas (e.g., greater cooperation and assertion reported by parents, greater social competence reported by teachers, higher liking by peers, for a review, see [11]). Congruently, a wide range of child psychiatric disorders are associated to impairments in facial emotion recognition, which are likely to negatively affect family and peer relationships (for a review, see [12]). For example, children with bipolar disorder or severe mood deregulation show deficits in labeling emotions—particularly negative emotions such as fear or anger—displayed by adult or child models [13]. This lower performance in emotion recognition tasks was also detected for abused or maltreated children (e.g., [14–16], for a review, see [17]).

Studies with children participants have frequently used facial expression databases depicting adults. For example, Barnard-Brak, Abby, Richman and Chesnut [18] have recently validated a sub-set of the NimStim [19] with a sample of very young children (2–6 years old), and showed that they can accurately label photographs of adults depicting happiness, sadness, anger and fear. Other studies used these materials to investigate whether the findings demonstrated with adult participants also generalize to children. For example, LoBue [20] also used pictures from the NimStim in a study related to emotion detection and showed that children share the attentional bias for angry faces (i.e., angry faces are detected faster than happy or neutral faces). A subsequent study using another database depicting adult models (KDEF; [21]) showed that negative facial expressions impaired children’s working memory to a greater extent, when compared to neutral and positive expressions [22].

Other studies have been using databases that include stimuli depicting non-adult models that can either be presented to children or adults. The availability of these databases is important for diverse research areas. In particular, these materials allow the use of peer-aged stimuli in studies with samples of children [23]. For example, a study with young children (3–5 years old) showed that the previously described attentional bias for angry faces is stronger when pictures of child (vs. adults) models are used [24]. Another important line of research did not focus on children’s responses, but rather on the behavioral [25,26] or psychophysiological responses of adults in general, or parents [27–29], to children’s emotional expressions. For example, Aradhye et al. [4] used photographs of children to examine how different expressions influence the responsiveness of non-kin young adults and found that smiling children were rated as more likely to be adopted than crying children. Other studies have even examined non-normative adult samples (e.g., maltreating parents or parents with psychiatric disorders). For instance, mothers with borderline personality disorder (vs. controls) showed an overall lower performance in recognizing emotion in children—both their own and unknown children—and to misinterpret neutral expressions as sadness [30]. Likewise, neglectful mothers [31] and abusive fathers [32] tend to perceive children’s emotional cues more negatively than non-maltreating parents.

Photographs of children’s facial expression can also be used to investigate how variables such as the age of the model influence person [33] or emotion [34] perception. For example, in a recent study by Griffiths, Penton-Voak, Jarrold, and Munafò [35], children and adult participants categorized the facial expressions of prototypes of different age groups (created by averaging photographs of individuals of the same gender and age group). Results showed similar accuracy for both child and adult facial expression prototypes across age groups. Thus, no evidence of own-age advantage emerged in either group of participants. Nevertheless, the age of the model did interact with other variables, such as gender (for a review, see [36]). For example, Parmley and Cunningham [34] showed that adult participants were more accurate to identify angry expressions displayed by male children than by female children, whereas no sex differences were detected in the identification of angry expressions displayed by adult models.

Currently, there are plentiful validated databases of facial expressions (for a review, see [37]). These databases include dynamic (i.e., videos) and static (i.e., pictures) stimuli depicting human models of different nationalities and cultural backgrounds, expressing a wide range of facial expressions. However, most databases include only young adults as models [19,21,37–39]. A few exceptions include adult models of distinct age groups. For example, the Lifespan Database of Adult Facial Stimuli [40] includes 18 to 93 years old models, and the FACES database [41] includes 19 to 80 years old models. As a consequence of this limited availability of validated databases depicting models across the lifespan, researchers often have to develop (and pre-test) new materials. For example, Parmley and Cunningham [34] selected a set of photographs of adults from existing databases, and complemented it with an original set of children’s photographs. In Table 1 we present an overview of the databases that include photographs of facial expressions of children (for dynamic stimuli databases, see for example [42,43]).

**Table 1:** Overview of children’s facial expressions databases.

	Stimuli			Validation Procedure	
Database	Image Set	Facial Expressions	Model Features	Sample/Country	Measures
Radbound Faces Database^a^ (RFD; [44])	1176 standardized color images: 240 of children, and 936 of adults	8 expressions: happiness, sadness, disgust, anger, fear, surprise, contempt, neutral Three gaze directions (left, frontal, right)	10 child models: 6 female 39 adult models: 19 female Specific age information not included. All Caucasian Dutch	276 adults (86% female, *M*_age_ = 21) All undergraduate students; Netherlands	Categorization of the expression (forced-choice: 7 Emotions + Neutral + Other) Subjective ratings of the expression (5-point scales): Intensity (*Weak* to *Strong*); Clarity (*Unclear* to *Clear*); Genuineness (*Faked* to *Genuine*); Valence (*Negative* to *Positive*) Subjective ratings of the model (only for the neutral, straight-gaze images, 5-point scale): Attractiveness (*Unattractive* to *Attractive*)
NIMH Child Emotional Faces Picture Set (NIMH-ChEFS; [45])	534 standardized color images	5 expressions: happiness, sadness, anger, fear, neutral Two gaze conditions (direct, averted)	60 models (child actors): 10–17 years old; 55 models Caucasian (categorization based on appearance)	20 adults (65% female, *M*_age_ = 38) All faculty / staff; USA	Categorization of the expression (forced-choice: 4 Emotions + Neutral + Other) Subjective rating of the expression (slider): Intensity (*Mild* to *Strong*); Representativeness (*Poorly* to *Very Well*);
Dartmouth Database of Children’s Faces (DDCF; [46])	640 standardized color images	8 expressions: happiness, content, sadness, disgust, anger, fear, surprise, neutral Five camera angles.	80 models: 6–16 years old; 40 female; All Caucasian	163 adults (59% female, *M*_age_ = 20) All undergraduate students; USA	Categorization of the expression (forced-choice: 6 Emotions + Neutral + Other) Subjective rating of the expression (5-point scales): Intensity (*Low intensity* to 5 *High intensity*); Age estimation of the model (in years)
Child Affective Facial Expression (CAFE; [2])	1192 standardized color images	7 expressions: happiness, sadness, disgust, anger, fear, surprise, neutral	154 models: 2–8 years old; 90 female; 77 Caucasian/ European American, 27 African- American, 23 Latino, 16 Asian, 11 South Asian	100 adults (50% female, *M*_age_ = 21) All undergraduate students; USA	Categorization of the expression (forced-choice: 6 Emotions + Neutral)
Child Emotions Picture Set (CEPS; [47])	225 standardized black and white images	7 expressions: happiness, sadness, disgust, anger, fear, surprise, neutral	17 models: 6–11 years old; 9 female; Multiracial backgrounds	30 experts (psychologists with experience in child development); Brazil	Categorization of the expression (forced-choice: 6 Emotions + Neutral) Categorization of the expression intensity: Weak (0%-30%); Moderate (31%-70%) or Strong (71%-100%)
White, Pardo and Black Children Picture Set (BIC-Multicolor; [48])	120 standardized color images	Neutral expression	120 models: 6–12 years old 66 female Race not predetermined	210 adults (71% female, *M*_age_ = 30); Brazil	Categorization of the model’s race (White, Pardo/Multiracial, Black) Subjective ratings (7-point scales): Facial Valence (*Negative* to *Positive*); Facial Friendliness (*Unfriendliness* to *Friendliness*)
Developmental Emotional Faces Stimulus Set^a^ (DEFSS; [23])	404 standardized color images: 144 of children, 154 of teens and 106 of adults	5 expressions; happiness, sadness, anger, fear, neutral	116 models: 42 children (8–12 years old), 44 teens (13–19 years old) and 30 adults 20–30 years old; 73 female, 102 White, 15 Non-White	228 participants: 20% children, 20% teens and 52% adults 75% female, 185 White, 39 Non-White; USA	Categorization of the expression (forced-choice: 4 Emotions + Neutral + None of the Above) Subjective ratings of the expression (7-point scale): Intensity (*Just a little* to *A lot*)
Tromsø Infant Faces (TIF; [49])	119 standardized color images	7 expressions: happiness, sadness, disgust, anger, fear, surprise, neutral	18 models: 4–12 months old; 10 female; All Caucasian	720 adults (79% female; *M*_age_ = 33) 50% with children; 90% from Norway or Germany	Categorization of the expression (forced-choice: 6 Emotions + Neutral + Other) Subjective ratings of the expression (5-point scales): Intensity (*Weak* to *Strong*); Clarity (*Ambiguous* to *Clear*); Valence (*Very Negative* to *Very Positive*)
City Infant Faces (CIF; [5])	195 naturalistic black and white images	3 expressions: negative, neutral, positive	68 models: 0–12 months old; 35 female; 62 Caucasian, 3 Asian, 2 Arab, 1 Indian	71 adults (89% female; *M*_age_ = 28) 58% midwives; 17% neonatal nurses; 25% general public; England	Categorization of the expression (forced-choice: Negative, Neutral, Positive) Subjective ratings of the expression (5-point scales): Intensity (*Weak* to *Strong*); Clarity (*Unclear* to *Clear*); Genuineness (*Fake* to *Genuine*); Affective response of the participant while viewing the image (forced choice: Negative, Neutral, Positive) and strength of response (*Weak* to *Strong*)
Youth Emotion Picture Set (YEPS; [50])	42 standardized black and white images	7 expressions: happiness, sadness, disgust, anger, fear, surprise, neutral	31 models: 12–20 years old; 14 male; 28 Caucasian, 1 Black, 3 Multiracial	101 adults (68% female); 54 adolescents (59% female); Brazil	Categorization of the expression (forced-choice: 6 Emotions + Neutral)
Baby Faces (BF; [51])	57 standardized color images	6 expressions; happiness, sadness, anger, fear, surprise, neutral	12 models: 6–12 months old; 6 female; 8 Caucasian, 2 Black, 2 Japanese	119 adults (64% female *M*_age_ = 36); Brazil	Categorization of the expression (forced-choice: 5 Emotions + Neutral)

*Note*. Number of pictures (and corresponding model description) refers to the stimuli used as materials for the validation procedure.

^a^ Database also includes images of adult models.

As shown in Table 1, nine databases exclusively with photographs of children’s facial expressions were recently published. These databases comprise standardized stimuli regarding graphic features (e.g., size, color, background) that were typically obtained through photoshoots in controlled settings (the CIF is an exception, with parents conducting the photoshoot and photographs processed by the authors). Facial expressions were prompted by employing different strategies during the photoshoot. For example, the models were exposed to videos (e.g., CEPS) or coached to imagine situations that would elicit the intended expression (e.g., “sitting on chewing gum” for eliciting disgust, DDCF). In other cases, the experience of the situation actually took place during the shoot (e.g., having infants tasting an unfamiliar food such as lemon to induce disgust, TIF). Despite these differences, all databases (except TIF and BIC-Multicolor) include specific emotions like happiness or anger, as well as neutral expressions. The characteristics of the models are also diverse across databases. For example, regarding age, the databases include photographs of infants (e.g., TIF; CIF; BF) or adolescent models (e.g., NIMH-ChEFS; DDCF). Nonetheless, there is a prevalence of Caucasian models across the databases (for exceptions, see [52,53]), which may limit the selection of ecologically valid stimuli in other cultural backgrounds (for a discussion on the implications of the demographic homogeneity of models, see [53]). Regarding the validation procedure, most studies were conducted with adult participants untrained in emotion recognition (an exception is the NIMH-ChEFS, which was subsequently validated with children and adolescents [54]), and typically entailed a forced-choice task to categorize the emotion depicted. In some cases, participants were also asked to rate the child expression in several evaluative dimensions (e.g., intensity, clarity, genuineness).

The CAFE [1,2] comprises the largest stimuli set (i.e., 1192 photographs) and is one of the most diverse databases regarding the race or ethnicity of the models, including Caucasian/European American, African-American, Latino, Asian, and South Asian children (see Table 1). The set includes a wide range of facial expressions—happiness, sadness, disgust, anger, fear, surprise, neutral–, with over 100 photographs per expression (minimum of 103 photographs depicting surprise, and maximum of 230 depicting a neutral expression). Another advantage of this database is the possibility to select different expressions produced by the same model. Moreover, although the models were photographed in constant conditions (e.g., same off-white background with overhead lighting), they are still depicted in a naturalistic way. For example, the hairstyle of the children is visible, in contrast with other databases such as the DDCF, which only shows the facial features and covers hair and ears.

The original CAFE stimuli were photographed by an expert (i.e., trained coder of facial expressions) and then validated by asking a sample of 100 untrained adult participants to identify the expressions (forced-choice task). As argued by Lobue and Trasher ([2], see also [19]), the use of untrained participants has the advantage of obtaining emotion recognition scores of participants who are similar to those who will be recruited in future studies. In the validation study, the overall accuracy rate was 66%. However, there were significant differences in accuracy across the seven facial expressions, with pictures depicting happiness obtaining the highest accuracy scores (85%), followed by surprise (72%), anger and neutral (66%), disgust (64%), sadness (62%), and fear (42%). These accuracy rates were all significantly different from each other (except for anger vs. neutral and disgust vs. sadness). Results also showed that emotion recognition accuracy was not systematically influenced by the characteristics of the model (i.e., sex and race/ethnicity). Regarding the characteristics of the participants, only a significant effect of sex emerged, such that women raters were more accurate than men at identifying all facial expressions.

A recent study examined preschoolers’ (3–4 years old) emotional recognition accuracy of a subset of the CAFE, and revealed strong associations between their ratings and those obtained in the original validation with adult participants [55]. Further corroborating the usefulness of this database, since its publication in 2015, the CAFE stimuli have been used as materials in multiple research domains, such as the neural processing of emotional facial expressions [28], attentional bias [24], stereotyping [56–59], and morality [60–62].

The racial/ethnic diversity of the models included in the CAFE makes it a particularly useful database for research in the stereotyping domain, namely to investigate if the racial biases identified in response to adults of specific social groups (e.g., Blacks) generalize to children of that same group. For example, in a sequential priming task, adult participants were faster to identify guns (vs. toys) when preceded by pictures of Black (vs. White) boys, suggesting that the perceived threat typically associated to Black men generalizes to Black boys [59]. Likewise, children expected the same negative event (e.g., biting their tongue) to induce less pain when experienced by Black (vs. White) children, demonstrating that the assumption that Back people feel less pain than White people also generalizes to Black children [56]. Importantly, by including children of different age groups as participants, this latter study also allowed to identify when such bias emerges in development, given that the effect was only strongly detected by the age of 10.

Our main goal was to further develop the CAFE database by assessing how the stimuli are perceived in a set of eight evaluative dimensions. Some of these dimensions require judgments about the model (i.e., familiarity, attractiveness, arousal, in-group belonging), whereas other are focused on the expression being displayed (i.e., valence, clarity, intensity and genuineness).

The measures regarding the facial expression have been assessed in other databases of children’s expressions (see Table 1). In contrast, the measures that entail judgments about the model are less common and have been assessed in validations of databases depicting adults (for a review, see [37]). For example, we included attractiveness ratings because attractive children (similar to attractive adults) are more positively perceived (e.g., more intelligent, honest, pleasant) than less attractive children (for a review, see [63]). Because the stimuli set was developed in a distinct cultural context we also included a measure of target’s in-group belonging (i.e., rating of the likelihood of the child being Portuguese). This measure can be of interest given the evidence that the recognition accuracy of facial expressions is higher when there is a match (vs. mismatch) between the cultural group of the expresser and of the perceiver (for reviews, see [64,65]). This in-group advantage for emotion recognition was also found with child participants when judging emotional expressions displayed by adults (e.g., [66]). Moreover, we also included a forced-choice expression recognition task to replicate the original validation study. The comparison of the accuracy scores obtained with our Portuguese sample with those produced by an American sample also informs about the cross-cultural validity of the database.

Lastly, we will also examine if individual factors (e.g., sex of the participant, parental status) impact emotion recognition and subjective ratings of the facial expressions. For example, it was shown that parents of young children rated images portraying facial expressions of infants as clearer, when compared with participants without children, or with older children (TIF database, [49]).

## Method

### Participants

The sample included 450 adult participants, from 18 to 71 years old (84.7% women; *M*_age_ = 32.34; *SD* = 10.76), of Portuguese nationality, who volunteered to participate in a web-survey. Regarding their ethnic/cultural background, most participants reported being of Portuguese ancestry (88.4%). The majority of participants were active workers (54.0%) or students (33.6%), who attained a bachelor’s degree (37.8%) or had completed high-school (36.4%). Regarding parental status, 43.8% of the participants were parents, and reported having up to four children (*M* = 1.66, *SD* = 0.76), with ages varying between 1 and 40 years old (*M*_age_ = 9.93, *SD* = 9.22).

### Materials

Our stimuli set included 283 images selected from CAFE [1]. The original database comprises color photographs of children posing in six basic emotional expressions (sadness, happiness, anger, disgust, fear and surprise), plus a neutral expression. The models (*N* = 154, 58.4% female) were heterogeneous in age (from 2 to 8 years old, *M*_age_ = 5.3) and ethnic background (50% Caucasian/European American, 17.5% African American, 14.9% Latino, 10.4% Asian and 7.1% South Asian). The models were prompted to display each of the emotions by the photographer, who exemplified the intended expression. All models were covered from the neck down with an off-white sheet. The final set of 1192 photographs corresponds to the number of poses deemed successful. The photographs are available in high resolution (2739 x 2739 pixels) and are standardized regarding background color (off-white), viewing distance and figure-ground composition.

The stimuli sub-set used in the current work was selected based on several criteria. First, we took into consideration the accuracy of emotional categorization (i.e., “proportion of 100 adult participants who correctly identified the emotion in the photograph”) reported in the original validation. Only photographs depicting facial expressions correctly identified by more than 50% of the sample were selected (resulting in 891 images). Second, we selected models that included photographs portraying neutral, happy and angry expressions (resulting in 455 images, 63 models). Third, we selected models that exhibited at least four different emotions (besides the neutral expression). Whenever different versions of the same emotion were available for the same model (e.g., happiness displayed with open and closed mouth), we selected the version that obtained the highest accuracy in the original database. Table 2 summarizes the characteristics of the photographs included in our sub-set (*N* = 283, corresponding to 51 models: 28 female, *M*_age_ = 4.81; 23 male, *M*_age_ = 5.00).

**Table 2:** Number of photographs for each emotional expression according to model’s race/ethnicity and model’s sex.

	African-American (13 models)		European (23 models)		Latino (8 models)		South Asian (4 models)		Asian (3 models)		
	F	M	F	M	F	M	F	M	F	M	*Total*
Emotion											
Anger	8	5	12	11	4	4	4	0	0	3	*51*
Neutral	8	5	12	11	4	4	4	0	0	3	*51*
Happiness	8	5	12	11	4	4	4	0	0	3	*51*
Disgust	6	4	8	10	4	3	3	0	0	2	*40*
Sadness	5	2	6	4	3	4	3	0	0	2	*29*
Fear	3	2	3	2	1	0	1	0	0	3	*15*
Surprise	8	5	11	10	3	4	2	0	0	3	*46*
*Total*	*46*	*28*	*64*	*59*	*23*	*23*	*21*	*0*	*0*	*19*	***283***

*Note*. F = Female model; M = Male model.

### Procedure

The study was reviewed and approved by the Ethics Committee of ISCTE-Instituto Universitário de Lisboa. The study involved human data collection from adult volunteers. The study was noninvasive, no false information was provided, data were analyzed anonymously and written informed consent was obtained. The use of CAFE stimuli was approved by the Ethics Committee of ISCTE-Instituto Universitário de Lisboa and consent was obtained from Databrary via the signature of an Access Agreement. The parents/guardians of the children participating in the original CAFE study [2] signed a release giving permission for the use of their data/image in scientific research.

Participants were invited (e.g., institutional email, social networking websites) to collaborate on a web-survey aimed at testing materials for future studies. The hyperlink directed participants to a secure webpage in Qualtrics. The opening page informed about the goals of the study (evaluation of photographs of children displaying different facial expressions), its expected duration (approximately 20 minutes), and ethical considerations (i.e., anonymity, confidentiality and the possibility to withdraw from the study at any point). After agreeing to collaborate in the study, participants were asked to evaluate each photograph considering their overall perception of the child portrayed (i.e., familiarity, attractiveness, arousal and likelihood of the child being Portuguese) as well as the facial expression displayed (i.e., valence, clarity, genuineness and emotional intensity). All evaluations were made in 7-point rating scales (for detailed instructions and scale anchors, see Table 3). In addition, participants were asked to identify the facial expression by selecting the corresponding label (i.e., sadness, happiness, anger, disgust, fear, surprise or neutral).

**Table 3:** Item wording and scale anchors for each dimension.

Dimension	Instructions: To what extent …	Scale Anchors
Model		
1. Attractiveness	… does this child look beautiful?	1 = *Not very beautiful*, 7 = *Very beautiful*
2. Arousal	… does this child look calm or excited?	1 = *Calm*, 7 = *Excited*
3. Familiarity	… does this child look familiar?	1 = *Not familiar at all*, 7 = *Very familiar*
4. In-group belonging	… is it likely that this child is Portuguese?	1 = *Certainly not Portuguese*, 7 = *Certainly Portuguese*
Expression		
5. Clarity	… is the expression displayed by the child clear?	1 = *Very unclear*, 7 = *Very clear*
6. Genuineness	… is the expression displayed by the child genuine?	1 = *Not genuine at all*, 7 = *Very genuine*
7. Intensity	… is the expression displayed by the child intense?	1 = *Not intense at all*, 7 = *Very intense*
8. Valence	… is the expression displayed by the child negative or positive?	1 = *Negative*, 7 = *Positive*

Participants were informed that there were no right or wrong answers. Instructions also emphasized that the presentation order of the evaluative dimensions would vary across photographs. Before initiating the evaluation task, participants were required to indicate their nationality (if other than Portuguese they were directed to the end of the survey), gender, current occupation and education.

To prevent fatigue and demotivation, participants were asked to rate a subset of 20 photographs. These photographs were randomly selected from the 283 available to minimize any systematic response bias deriving from the composition of the subsets. Each trial corresponded to the evaluation of one photograph. Specifically, in a single page of the web-survey, the image was presented at the center of the page with all the rating scales below it. The rating scales were presented in a random order across trials. However, the facial expression identification task (labeling) was always presented at the end of each trial. The seven emotional labels were also presented in a random order across trials.

At the end of the 20 trials, participants were asked to report their cultural background (i.e., Portuguese of… “Portuguese ancestry”, “African ancestry”, “Brazilian ancestry”; “Ukrainian ancestry” or “Other”), as well as their parenting status (parents were also asked to report the number of children, as well as the age of each child). Finally, participants were asked if their work entails regular contact with children, and if they have social contact with children other than their own (both using the following scale anchors: 1 = *No regular contact at all*; 7 = *Very regular contact*). Upon completion of the questionnaire, participants were thanked and debriefed.

## Results

Given that we only retained completed questionnaires for analyses (*N* = 450) there were no missing cases. The preliminary analysis of the data showed no indication of systematic responses (i.e., participant using the same value of the response scale across dimensions) and a small percentage of outliers (1.02%—outliers were identified considering the criterion of 2.5 standard deviations above or below the mean evaluation of each stimulus in a given dimension). Therefore, no responses were excluded.

Below, we will present the analyses required to validate the stimulus set, as well as additional analyses that are potentially useful for researchers interested in using the set:

1. Overall subjective ratings: We present the descriptive statistics of the subjective ratings for the entire sample and compare ratings according to participants’ gender and parental status. Additionally, we also examined the associations between evaluative dimensions and examined the role of individual differences (e.g., age, frequency of contact with children in social and work contexts) in these associations.
2. Impact of facial expression and model characteristics on subjective ratings: We compared ratings across evaluative dimensions according to facial expression (i.e., sadness, happiness, anger, disgust, fear, surprise or neutral), and model characteristics (i.e., sex and race/ethnicity of the model);
3. Emotion recognition: We examined individual differences in overall accuracy. We also examined the impact of the expression, as well as the influence of model characteristics, on the accuracy of emotion recognition (mean % of hit rates);
4. Cross-cultural comparison: We compared the accuracy in emotion recognition between the original and the current validation according to emotion type;
5. Frequency distribution: To facilitate the overall characterization of the stimuli in the set we also present the frequency distribution of images across three levels (low, moderate and high) of each evaluative dimension.

Each photograph was evaluated by a minimum of 31 and a maximum of 34 participants. Normative and raw data files are available at https://osf.io/mjqfx/. Appendix A includes item level data (i.e., descriptive results for the set of eight evaluative dimensions and accuracy rates of emotion recognition. Each photograph is described (e.g., file name, model characteristics and facial expression) according to the original CAFE database. Appendix B comprises normative data organized by participant (including socio-demographic information of the raters), overall emotion accuracy rate, and ratings for each evaluative dimension according to facial expression, and model’s characteristics (i.e., sex and race/ethnicity). Appendix C includes full raw data.

### Overall subjective ratings

We compared ratings across evaluative dimensions against the scale midpoint and tested for gender and parental status differences considering the entire set of stimuli (see Table 4).

**Table 4:** Mean evaluations across dimensions (entire sample; for women vs. men; for parents vs. non-parents).

Dimension	Total (*n* = 450)		Women (*n* = 381)		Men (*n* = 69)		Difference Test			Parents (*n* = 253)		Non-parents (*n* = 197)		Difference Test		
	*M*	*SD*	*M*	*SD*	*M*	*SD*	*t*(449)	*p*	*d*	*M*	*SD*	*M*	*SD*	*t*(448)	*p*	*d*
Familiarity	3.94	1.01	3.96	1.00	3.84	1.03	1.31	.191	0.12	3.79	1.02	4.14	0.96	3.64	< .001	0.34
Attractiveness	4.81*	0.89	4.89	0.86	4.39	0.91	5.98	< .001	0.56	4.75	0.89	4.90	0.89	1.78	.077	0.17
Arousal	4.25*	0.57	4.27	0.59	4.15	0.48	2.29	.023	0.22	4.30	0.54	4.19	0.61	-2.05	.041	0.19
In-Group	3.73*	0.67	3.79	0.67	3.41	0.62	6.25	< .001	0.59	3.70	0.62	3.77	0.74	1.11	.266	0.10
Valence	3.78*	0.53	3.79	0.52	3.71	0.58	1.67	.096	0.16	3.77	0.54	3.80	0.51	< 1	.550	0.06
Clarity	4.97*	0.72	4.97	0.73	4.98	0.71	< 1	.889	0.01	4.97	0.70	4.98	0.76	< 1	.914	0.01
Genuineness	4.82*	0.73	4.83	0.73	4.74	0.76	1.20	.233	0.11	4.78	0.70	4.87	0.77	1.31	.192	0.12
Intensity	4.88*	0.62	4.91	0.62	4.74	0.60	2.95	.003	0.28	4.83	0.59	4.95	0.65	1.98	.049	0.19

Note.

*Different from scale midpoint (i.e., 4).

Means and standard deviations are weighted to follow Portuguese male and female population effectives (weighting factors: Females = 0.617; Male = 3.123).

Overall, participants evaluated the photographs above the scale midpoint in attractiveness, arousal, clarity, genuineness and intensity, and below the scale midpoint for in-group belonging and valence, all *p*s ≤ .001. Familiarity ratings did not differ from the scale midpoint, *p* = .241. Regarding gender differences, results show that women provided higher attractiveness, arousal, in-group belonging, and intensity ratings than men. Lastly, parents evaluated the stimuli as more familiar, more intense, and aroused than non-parents.

The correlations between evaluative dimensions are described in Table 5. Taking the strength of the correlation as criteria [67], we report correlations that were at least weak (i.e., *r* ≥ .20). Results showed that clarity was strongly and positively associated with both genuineness and with intensity, such that facial expressions rated as clearer were also perceived as more genuine and intense. We also found a strong and positive association between genuineness and intensity. Familiarity ratings showed a moderate positive correlation with in-group belonging (i.e., models rated as more familiar were also perceived as more likely to be Portuguese). We also found the same type of correlation between intensity and arousal (i.e., children displaying more intense expressions were also perceived as more aroused). Attractiveness ratings were only weakly and positively associated with the remaining evaluative dimensions, as were the associations between arousal and clarity and genuineness, and between genuineness and familiarity and valence.

**Table 5:** Correlations.

	1	2	3	4	5	6	7	8	9	10
1. Familiarity	-									
2. Attractiveness	.30***	-								
3. Arousal	.06	.20***	-							
4. In-group	.49***	.30***	.09	-						
5. Valence	.18***	.20***	.02	.18***	-					
6. Clarity	.19***	.28***	.23***	.07	.16***	-				
7. Genuineness	.27***	.38***	.24***	.11*	.22***	.66***	-			
8. Intensity	.19***	.33***	.40***	.11*	.12**	.69***	.64***	-		
9. Contact: Work	.19***	.10*	.02	.08	.01	.03	-.01	.05	-	
10. Contact: Social	.11*	.18***	-.05	.08	.09	-.01	.05	.01	.38***	-
11. Age	.17***	0.04	-.09	.02	.04	.03	.08	.12*	.27***	.24***

*** *p* ≤ .001;

** *p* ≤ .010;

* *p* ≤ .050

Frequency of contact with children in a work context was weakly and positively correlated with frequency of contact in a social context, and both variables were also weakly associated with participants’ age. Note that overall the associations between these variables and the subjective ratings were non-significant or very weak (i.e., associations between each of these variables and familiarity, as well between frequency of work and social contact and attractiveness).

### Impact of facial expression and model characteristics on subjective ratings

We computed mean ratings for each of the 283 stimuli across the eight evaluative dimensions and conducted three separate univariate ANOVAs to examine the influence of facial expression, the sex and race/ethnicity of the model on each variable (post-hoc comparisons were conducted with Bonferroni correction and only the extreme values will be presented). Descriptive results (means and standard deviations) are summarized in Table 6.

**Table 6:** Hit rates (%) and subjective ratings according to emotion, sex of the model and model’s race/ethnicity.

	Hit Rate (%)		Familiarity		Attractiveness		Arousal		In-group		Valence		Clarity		Genuineness		Intensity		
	*M*	*SD*	*M*	*SD*	*M*	*SD*	*M*	*SD*	*M*	*SD*	*M*	*SD*	*M*	*SD*	*M*	*SD*	*M*	*SD*	*N*
Emotion																			
Anger	78.74	16.35	3.85	0.37	4.61	0.46	5.26	0.54	3.76	0.59	2.29	0.37	5.26	0.56	4.62	0.51	5.68	0.54	*51*
Disgust	69.46	21.33	3.86	0.38	4.60	0.44	4.54	0.50	3.76	0.64	2.91	0.55	4.84	0.62	4.71	0.51	5.09	0.50	*40*
Fear	58.43	15.89	3.91	0.40	4.81	0.42	4.62	0.46	3.72	0.72	2.92	0.36	5.06	0.53	4.97	0.33	5.30	0.45	*15*
Happiness	89.01	13.82	4.14	0.44	5.03	0.52	4.07	0.56	3.72	0.56	5.99	0.45	5.47	0.59	5.18	0.66	4.93	0.63	*51*
Neutral	74.80	17.96	3.75	0.40	4.88	0.50	2.67	0.32	3.60	0.64	3.73	0.50	3.93	0.38	4.83	0.31	3.37	0.48	*51*
Sadness	70.10	22.73	3.88	0.37	4.67	0.44	3.79	0.45	3.77	0.58	2.36	0.33	4.87	0.71	4.26	0.77	4.68	0.60	*29*
Surprise	79.87	16.59	4.17	0.39	4.99	0.45	4.99	0.64	3.83	0.57	4.98	0.84	5.41	0.50	5.00	0.60	5.44	0.55	*46*
Model Sex																			
Female	75.57	18.82	3.97	0.41	4.85	0.51	4.20	1.02	3.75	0.54	3.69	1.46	4.95	0.74	4.82	0.60	4.88	0.93	*154*
Male	78.25	19.65	3.91	0.43	4.76	0.48	4.31	1.01	3.72	0.67	3.88	1.47	5.00	0.80	4.80	0.63	4.89	0.97	*129*
Model Race/Ethnicity																			
African	77.40	15.62	4.03	0.36	4.93	0.41	4.26	1.02	3.69	0.40	3.92	1.51	4.99	0.77	4.92	0.69	4.91	0.99	*74*
Asian	73.51	18.34	4.01	0.44	4.74	0.32	4.31	1.00	3.84	1.08	3.67	1.37	5.02	0.80	4.83	0.64	4.93	1.10	*19*
European	78.12	20.22	3.91	0.44	4.89	0.56	4.29	1.02	3.69	0.56	3.75	1.47	5.04	0.72	4.79	0.53	4.91	0.94	*123*
Latino	75.06	20.94	3.91	0.42	4.63	0.39	4.18	1.06	3.86	0.64	3.84	1.46	4.81	0.89	4.76	0.70	4.80	0.92	*46*
South Asian	73.65	22.22	3.83	0.48	4.39	0.45	4.10	0.97	3.77	0.74	3.42	1.41	4.83	0.72	4.69	0.46	4.83	0.81	*21*
*Total*	*76*.*79*	*19*.*22*	*3*.*94*	*0*.*42*	*4*.*81*	*0*.*50*	*4*.*25*	*1*.*01*	*3*.*73*	*0*.*60*	*3*.*78*	*1*.*47*	*4*.*97*	*0*.*77*	*4*.*81*	*0*.*61*	*4*.*89*	*0*.*94*	*283*

#### Familiarity

Familiarity ratings varied according to the type of facial expression, *F*(1,6) = 7.53, *MSE* = 1.27, *p* < .001, η_p_^2^ = .14. Photographs displaying surprise obtained the highest familiarity ratings, all *p*s ≤ .008 (but not different from sadness, *p* = .053, fear, *p* = .617 and happiness, *p* = 1.000), and neutral photographs obtained the lowest familiarity ratings, all *p*s < .001 (but not different from anger, disgust, fear and sadness, all *p*s = 1.000).

Familiarity ratings did not vary according to model’s sex, *F*(1,281) = 1.76, *MSE* = 0.31, *p* = .186, η_p_^2^ = .01, or race/ethnicity, *F*(4,278) = 1.57, *MSE* = 0.28, *p* = .182, η_p_^2^ = .02.

#### Attractiveness

Attractiveness ratings also varied according to facial expression, *F*(1,6) = 6.69, *MSE* = 1.49, *p* < .001, η_p_^2^ = .13. Photographs displaying happiness obtained the highest attractiveness ratings, all *p*s ≤ .019 (but not different from fear, neutral and surprise, all *p*s = 1.000), and those displaying disgust obtained the lowest attractiveness ratings, all *p*s ≤ .002 (but not different from anger, fear, neutral and sadness, all *p*s > .099).

Attractiveness ratings did not vary according to the sex of the model, *F*(1,281) = 2.61, *MSE* = 0.65, *p* = .107, η_p_^2^ = .01. However, results show the impact of model’s race/ethnicity on attractiveness ratings, *F*(4,278) = 7.96, *MSE* = 1.80, *p* < .001, η_p_^2^ = .10. Specifically, African-American models obtained the highest attractiveness ratings, all *p*s ≤ .007 (but not different from Asian and European, both *p*s = 1.000) and South Asian models obtained the lowest attractiveness ratings, all *p*s < .001 (but not different from Asian, *p* = .216, and Latino, *p* = .602).

#### Arousal

Arousal ratings varied according to facial expression, *F*(1,6) = 136.66, *MSE* = 36.13, *p* < .001, η_p_^2^ = .75. Specifically, we observed that models displaying anger were perceived as more aroused, all *p*s ≤ .001 (but not different from surprise, *p* = .214), and that those with neutral expressions obtained the lowest arousal ratings, all *p*s < .001.

Arousal ratings did not vary according to the sex, *F* < 1, or the model’s race/ethnicity, *F* < 1.

#### In-group belonging

Ratings regarding the likelihood of the model being Portuguese did not vary according to the emotion displayed, the sex or the model’s race/ethnicity, all *F* < 1.

#### Valence

Valence ratings varied according to facial expression, *F*(1,6) = 311.80, *MSE* = 87.94, *p* < .001, η_p_^2^ = .87, such that photographs displaying happiness were rated as the most positive, all *p*s < .001, and that photographs displaying anger were rated as the most negative, all *p*s ≤ .002 (but not different from sadness, *p* = 1.000).

Valence ratings did not vary according to the sex, *F*(1,281) = 1.22, *MSE* = 2.61, *p* = .271, η_p_^2^ = .00, or the model’s race/ethnicity, *F* < 1.

#### Clarity

Clarity ratings varied according to the facial expression, *F*(1,6) = 44.64, *MSE* = 13.62, *p* < .001, η_p_^2^ = .49. Specifically, happiness was perceived as the clearest expression, all *p*s < .001 (but not different from fear, *p* = .258, anger and surprise, both *p*s = 1.000), and neutral photographs were rated as the least clear, all *p*s < .001.

Clarity ratings did not vary according to the sex of the model or its race/ethnicity, both *F* < 1.

#### Genuineness

Genuineness ratings varied according to facial expression, *F*(1,6) = 11.09, *MSE* = 3.38, *p* < .001, η_p_^2^ = .19, with photographs displaying happiness perceived as the most genuine, all *p*s ≤ .031 (but not different from fear and surprise, both *p*s = 1.000), and photographs displaying sadness rated as the least genuine, all *p*s ≤ .016 (but not different from anger, *p* = .112).

Genuineness ratings did not vary according to the sex of the model, or its race/ethnicity, both *F* < 1.

#### Intensity

Intensity ratings varied according to facial expression, *F*(1,6) = 94.94, *MSE* = 28.19, *p* < .001, η_p_^2^ = .67, with photographs displaying anger perceived as the most intense, all *p*s < .001 (but not different from fear, *p* = .354 and surprise, *p* = .623), and neutral photographs rated as the least intense, all *p*s < .001.

Intensity ratings did not vary according to the sex or race/ethnicity of the model, both *F* < 1.

Overall, we observed differences across subjective ratings according to the type of emotional expression, but not according to the characteristics (sex, race/ethnicity) of the models.

### Facial expression recognition

Hit scores (%) were obtained for each stimulus by calculating the percentage of participants that correctly recognized the intended expression based on the number of participants that evaluated a given photograph.

Results showed that the mean accuracy rate across the full set of 283 photographs was 76.8%. No differences were found according to the sex of the rater—women (*M* = 77.01%, *SD* = 12.69) and men (*M* = 75.51%, *SD* = 11.10), *t*(449) = 1.33, *p* = .184, *d* = 0.13. Surprisingly, participants without children (*M* = 78.77%, *SD* = 11.51) were more accurate than those with children (*M* = 74.21%, *SD* = 13.17), *t*(448) = 3.92, *p* < .001, *d* = 0.37. However, when examining the accuracy levels of those who reported having younger children (i.e., up to 8 years old—the maximum age of the models), parents with at least one young child were significantly more accurate (*M* = 76.64%, *SD* = 11.62) than parents with older children (*M* = 69.59%, *SD* = 15.53), *t*(187) = 3.49, *p* = .001, *d* = 0.51.

We also examined the influence of facial expression, and both sex and race/ethnicity of the model by conducting three separate univariate ANOVAs (see Table 6). As expected, accuracy varied according to the facial expression, *F*(1,6) = 8.94, *MSE* = 2824.85, *p* < .001, η_p_^2^ = .16 (see Table 6). Post-hoc comparisons with Bonferroni correction, showed that photographs displaying happiness obtained the highest accuracy rates, all *p*s ≤ .001 (but not different from anger, *p* = .080, and surprise, *p* = .252), and that photographs displaying fear obtained the lowest accuracy rates, all *p*s ≤ .040 (but not different from sadness, *p* = .839, and disgust, *p* = .869). Accuracy rates did not vary according to the sex, *F*(1,281) = 1.37, *MSE* = 505.15, *p* = .243, η_p_^2^ = .01, or the model’s race/ethnicity, *F* < 1.

Again, we observed differences on accuracy rates according to the type of expression, but not according to the models’ characteristics.

### Cross cultural comparison

To compare the mean accuracy rates observed in our sample (for the same sub-set of stimuli) with those reported in the original validation study [2], we conducted a 2 (sample) x 7 (facial expression) univariate ANOVA.

**Fig 1:**
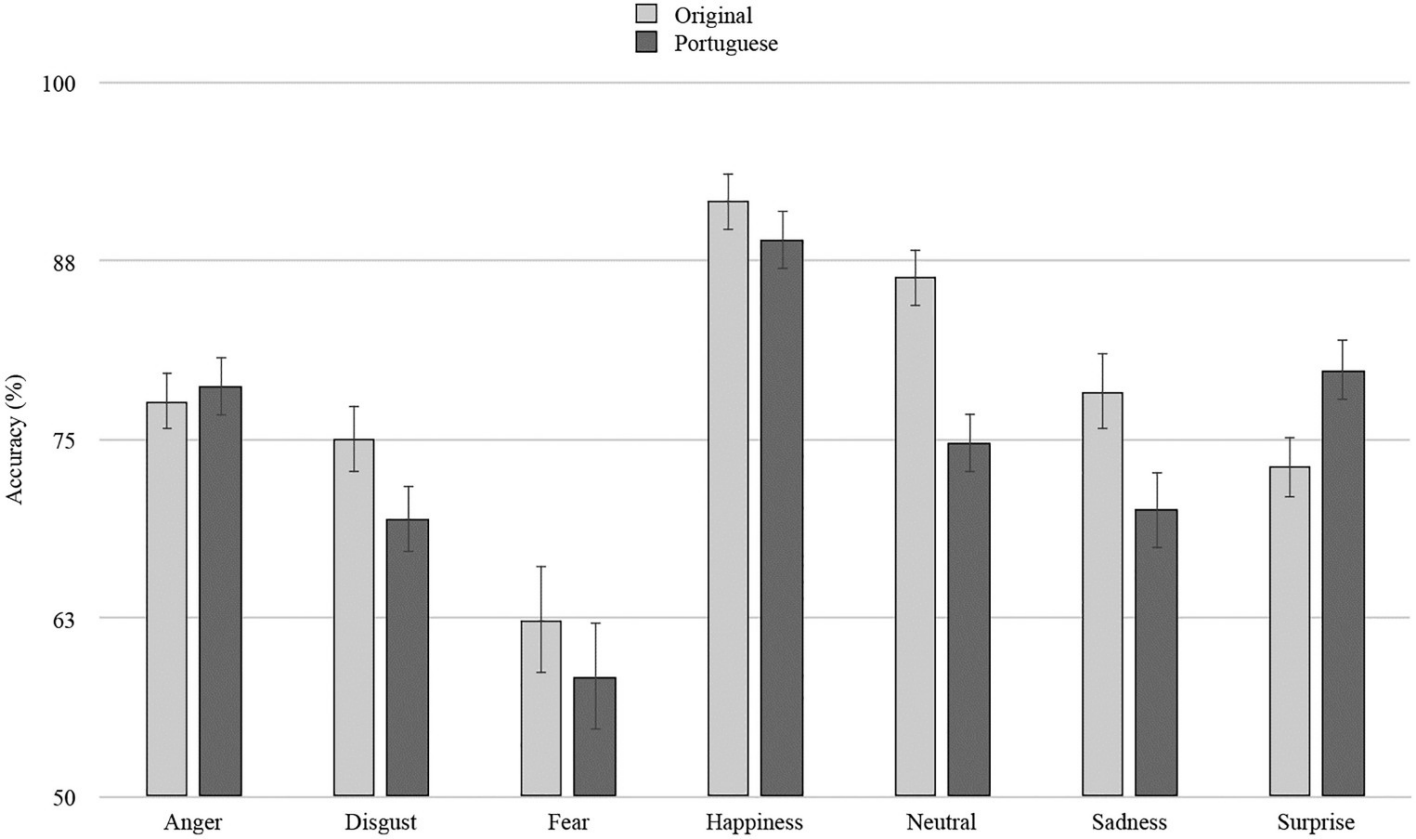
Comparison of mean accuracy rates (%) between samples by facial expression.

Results showed a main effect of sample, *F*(1,552) = 6.87, *MSE* = 1422.80, *p* = .009, η_p_^2^ = .01, such that the accuracy rates observed with the Portuguese sample (*M* = 74.3%, *SE* = .94) were lower than the ones reported in the original validation sample (*M* = 77.8%, *SE* = .94). We also observed a main effect of emotion, *F*(6,552) = 23.40, *MSE* = 4849.70, *p* < .001, η_p_^2^ = .20, such that photographs displaying happiness obtained the highest accuracy rates, all *p*s < .001, and photographs displaying disgust obtained the lowest accuracy rates, all *p*s ≤ .003 (but not different from anger, *p* = .121, sadness and disgust, both *p*s = 1.000). Moreover, results showed an interaction between sample and facial expression, *F*(6,552) = 4.03, *MSE* = 835.20, *p* = .001, η_p_^2^ = .04 (see Fig 1).

As shown in Fig 1, the original validation (vs. Portuguese) obtained higher accuracy ratings for neutral stimuli, *t*(552) = 4.05, *p* < .001, *d* = 0.34, as well as for those depicting sadness, *t*(552) = 2.19, *p* = .029, *d* = 0.19. For surprise, higher accuracy was observed in the current validation, *t*(552) = -2.25, *p* = .025, *d* = 0.19. No differences between samples were observed for the remaining expressions, all *p*s > .083.

### Frequency distribution

**Fig 2:**
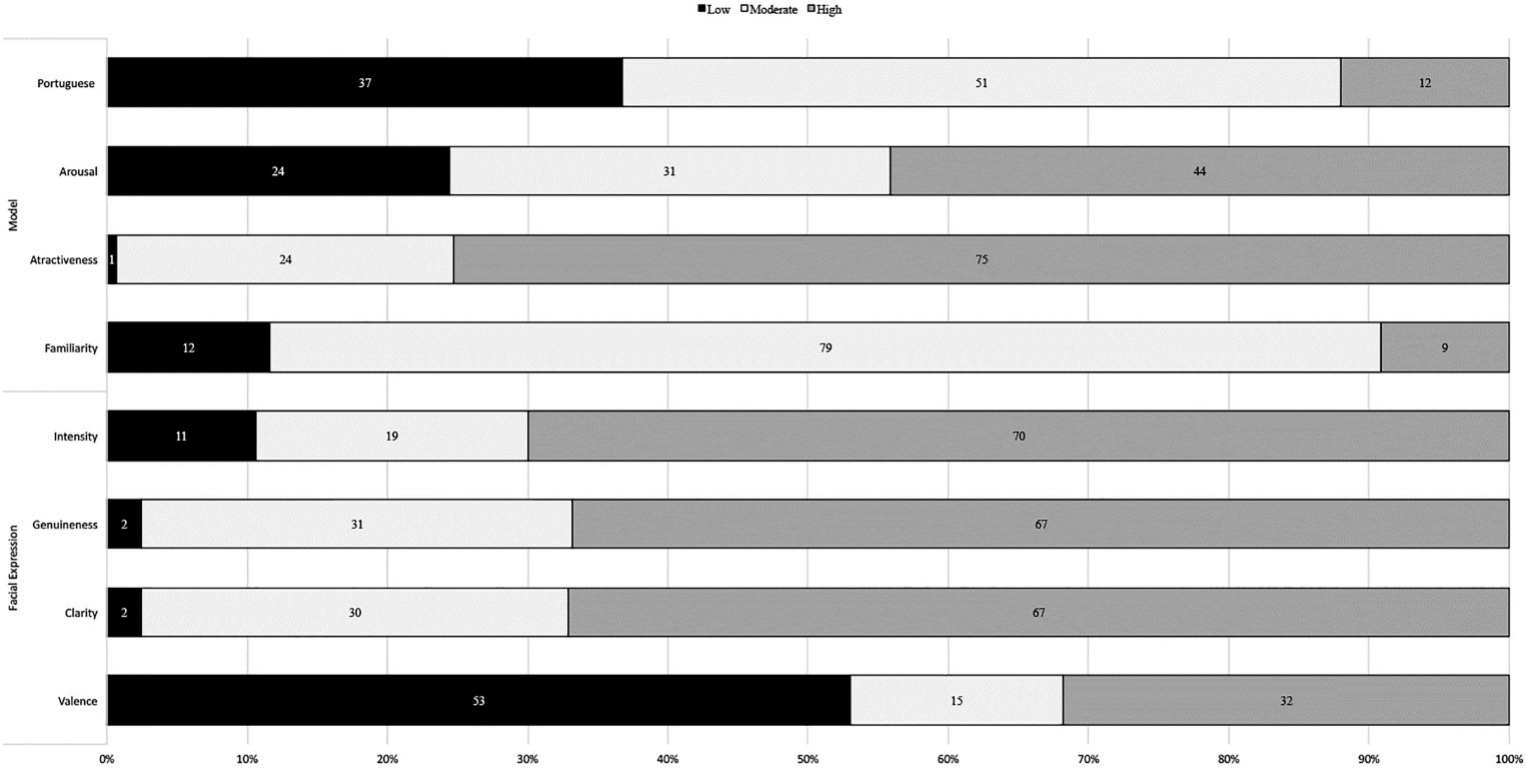
Distribution of photographs across each dimension level.

We computed descriptive statistics (i.e., means, standard deviations and confidence intervals) for each photograph per evaluative dimension (see https://osf.io/mjqfx/). According to the confidence interval, each photograph was categorized as low (i.e., lower bound below scale midpoint), moderate (confidence interval included the scale midpoint) or high (lower bound above scale midpoint) on a given dimension (for a similar procedure, see [68–70]. For the valence dimension, the low, moderate and high levels correspond to negative, neutral and positive, respectively. Fig 2 represents the frequency distribution of photograph across dimensions.

Regarding the evaluative dimensions concerning the model, results showed that most photographs were perceived as moderate in familiarity (79%) and in likelihood to belong to the in-group (51%), and as high in attractiveness (75%). In the case of arousal, photographs were distributed across the three levels with the highest percentage of photographs evaluated as high in arousal. Regarding the dimensions related to the evaluation of the expression, most photographs were perceived as high in intensity (70%), genuineness (67%) and clarity (67%), and also as negative (53%).

## Discussion

Databases of children’s facial expressions have been used in a myriad of research domains, such as emotion detection and recognition, social cognition (e.g., impression formation, stereotypes), cognitive psychology (e.g., attention bias), with samples of normative or non-normative (e.g., psychiatric disorders) children or adults (parents or non-parents).

In this work, we provide further validation for a sub-set of one of the most comprehensive databases of facial expressions depicting children—the CAFE [2]. This sub- set (283 photographs) is varied regarding the characteristics of the model, as it includes stimuli depicting boys and girls of heterogeneous race/ethnicity. It is also varied in the range of expressions depicted (i.e., sadness, happiness, anger, disgust, fear, surprise, neutral). Moreover, one of the primary criteria for selecting stimuli for the current validation was to select models that exhibited at least four different emotions (51 models)—with angry, neutral and happy expressions mandatory. Angry and happy faces have been used to activate negative versus positive valence (e.g., [71]), or as exemplars of socially aversive versus appetitive stimuli (e.g., [72]). The availability of neutral expression for all the models is also of particular interest, as these stimuli may serve as baseline in several experimental paradigms (e.g., affective priming, approach-avoidance tasks), or as the target stimuli in impression formation tasks (e.g., [73]). Besides assessing emotion recognition accuracy (as in the original validation), we also asked participants to evaluate each stimulus in eight subjective dimensions focusing on the characteristics of the model or of the expression depicted.

Based on the overall mean ratings, the facial expressions were rated as high in clarity, genuineness and intensity, and the models were perceived as high in attractiveness and arousal, as moderately familiar and as low in their likelihood of in-group belonging. Overall valence ratings were negative, which is not surprising considering the range of facial expressions included (i.e., fear, sadness, anger and disgust vs. happiness, surprise and neutral). Differences according to the sex of the rater were only found for a few dimensions, such that woman (vs. men) evaluated the models as more attractive, aroused and as more likely to belong to the in-group, and the expressions as more intense. Parental status also impacted mean ratings, such that parents (vs. non-parents) evaluated the models as more familiar and less aroused, and the expressions as more intense.

The overall accuracy in emotion recognition was satisfactory (77%) and did not vary according to the sex of the rater. This finding contrasts with the results from the original validation CAFE validation (i.e., higher accuracy rates for female respondents), but is in line with the results obtained in other validations of children’s photos (e.g., [49]). Parental status did impact overall accuracy, but in the reverse direction: overall non-parents were actually more accurate than parents. However, parents of younger children (up to 8 years old, as the models included in our sub-set) were more accurate than those with older children. Previous studies that examined parental status have also failed to demonstrate a general advantage of parents in children’s emotion recognition (e.g., [49]). In turn, differences regarding parental status seem to be found only in interaction with other variables, such as sex and type of facial expression [26]. Finally, the overall ratings were not strongly associated with the frequency of contact with children (both in work and social contexts).

Accuracy also varied according to the facial expression, with the highest accuracy rate obtained for happy faces (although not statistically different from anger and surprise). Indeed, studies have consistently shown an advantage in the recognition speed and/or accuracy of happy faces in comparison to other basic emotional categories (for a review, see [74]). The accuracy of emotion recognition was independent of the models’ characteristics such as sex or race/ethnicity, replicating the original CAFE validation. Finally, the comparison of the results of the emotional recognition measure between our sample and the original validation for the same sub-set of stimuli, showed that overall, the accuracy rates of the Portuguese sample were lower. However, this difference was inferior to 4% and was due to higher recognition rates for neutral and sad faces in the original sample. Indeed, the accuracy rates for faces depicting surprise were higher in the Portuguese sample, whereas no cross-cultural differences were detected for the other facial expressions.

Overall, we found positive correlations between most evaluative dimensions (e.g., clarity was strongly and positively associated with genuineness and with intensity and the latter dimensions were also strongly associated). Importantly, the impact of facial expression was found for all dimensions (except judgments of in-group belonging). For example, happy faces were perceived as the most attractive, positive, clear and genuine, whereas angry faces were rated as the most aroused and intense. The characteristics of the models (i.e., sex, race/ethnicity) did not impact these ratings. Indeed, the only effect regarding race/ethnicity detected was for the attractiveness dimension, with African models rated as the most attractive (along with Asian and European models).

The CAFE database is suitable to be used with adult participants (e.g., to study how normative and non-normative samples differ regarding emotion recognition of child facial expressions). Moreover, this database is particularly useful in research conducted with samples of children as it allows for the use of peer-aged stimuli. Yet, the generalization of the current norms to children should be made cautiously. Although no differences between child and adult raters have been reported regarding emotion recognition performance [55], that might not be the case for some of the subjective dimensions. For example, a recent study showed that although ratings of valence and arousal produced by adults and children regarding facial expressions depicted by adult models were correlated, some differences emerged according to the raters’ age group (e.g., children rated all expressions more positively [75]). The replication of the current validation procedure with children is recommended.

In sum, the current CAFE sub-set is diverse regarding the objective characteristics of the models and the range of facial expressions depicted. Note however, that this sub-set is limited regarding certain emotional expressions (e.g., photographs of fear expression are only available for 15 models). Another limitation is that the several model characteristics (race/ethnicity, sex and emotional expression) are not fully balanced (e.g., South Asian models are all females and Asian models are all males). This imbalance derives both from the distribution of exemplars across all categories in the original database and from the criteria used to select the subset for the current study. Also, the choice is limited for researchers interested in ambiguous facial expressions, as only 35 photographs show recognition rates below 50%. We expanded the original database by assessing an extensive set of evaluative dimensions. Most stimuli were rated as depicting genuine, clear and intense facial expressions. Also, regarding the evaluation of the models, most stimuli were evaluated as portraying familiar and attractive children. Results from the in-group belonging measure suggest the applicability of this set across different cultural backgrounds. For example, Portuguese participants indicated that most pictures (63%) depicted models with a moderate or high likelihood of belonging to their in-group. For valence and arousal dimensions, the stimuli are more equally distributed across the three levels of the dimensions. Hence, numerous exemplars of each level can be selected for future research. This normative data allows researchers to select adequate stimuli according to different criteria, for example manipulating the dimensions of interest (e.g., type of expression), while controlling for other variables (e.g., model characteristics).
